# P-340. Adaptation of a Pharmacist-Led Long-Acting Injectable (LAI) Antiretroviral Therapy (ART) Program to Increase Use of LAI ART in Arkansas, an Ending the HIV Epidemic (EHE) Jurisdiction

**DOI:** 10.1093/ofid/ofaf695.558

**Published:** 2026-01-11

**Authors:** Amy L Brotherton, Jure Baloh, Azizi Ray, Gabriella A Douglass, Diane Ayuninjam, Aurielle M Thomas, Ashlyn Curry, Elma Abdullah, Dewansia Sutton, Jesse D Moore, Sarah A Marshall, Curt Beckwith

**Affiliations:** The Miriam Hospital, Brown University Health;Warren Alpert Medical School of Brown University, Providence, RI; University of Arkansas for Medical Sciences, Little Rock, Arkansas; University of New Mexico Heath Sciences Center, Albuquerque, NewMexico; ARcare, Searcy, Arkansas; The Miriam Hospital, Brown University Health, Providence, Rhode Island; The Miriam Hospital, Brown University Health, Providence, Rhode Island; University of Arkansas for Medical Sciences, Little Rock, Arkansas; ARcare, Searcy, Arkansas; ARcare/MississippiCare, Oxford, Mississippi; ARcare, Searcy, Arkansas; University of Arkansas for Medical Sciences, Little Rock, Arkansas; Warren Alpert Medical School of Brown University, Providence, Rhode Island

## Abstract

**Background:**

Barriers to adherence to daily oral ART can lead to poor clinical outcomes and propagation of HIV transmission. LAI ART is a promising alternative for persons with HIV (PWH) who struggle with adherence or prefer a non-oral option. Access to LAI ART for PWH has been complicated by administrative, clinical, and logistical barriers. The Miriam Hospital Infectious Diseases & Immunology Center (TMH ID Center) in Providence, Rhode Island established a successful pharmacist-led LAI ART program overcoming implementation barriers. This study sought to adapt and pilot a similar program at ARcare, a Ryan White-funded Federally Qualified Health Center (FQHC) with multiple HIV clinics in Arkansas, an EHE-designated jurisdiction.

**Figure:**
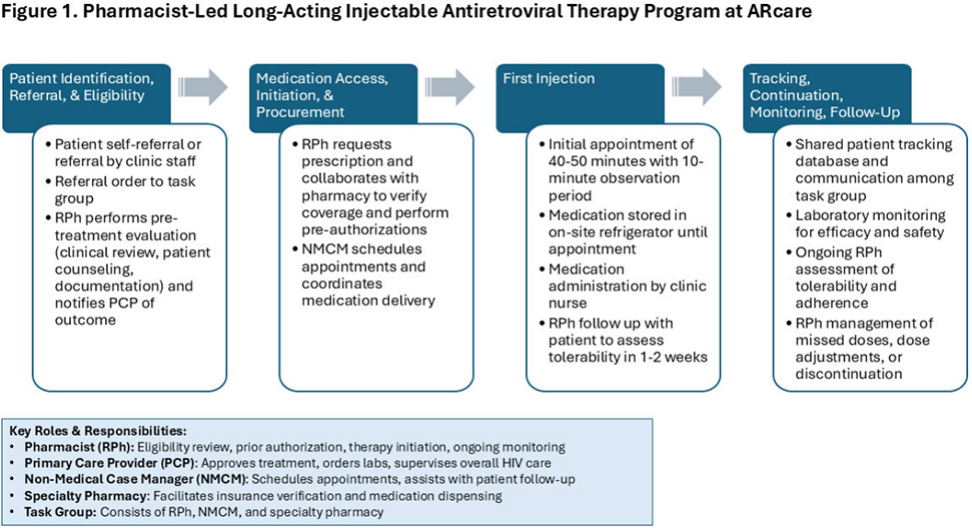

**Figure:**
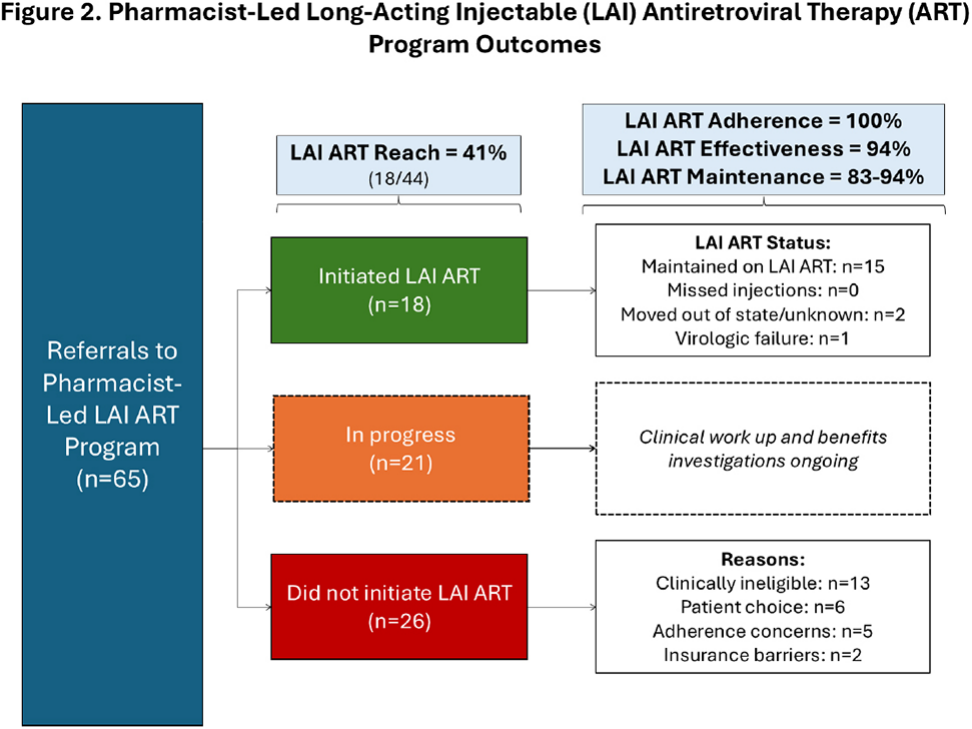

**Methods:**

This was a two-year study supported by the Providence/Boston Center for AIDS Research (P30AI042853). Leveraging the procedures and insights from the TMH ID Center LAI ART Program, we adapted a pharmacy-led LAI ART program (Figure 1) through the development of a Collaborative Practice Agreement (CPA) between pharmacists and providers across ARcare clinics. Implementation was supported by weekly meetings between ARcare and TMH to discuss clinical and logistical challenges. Guided by the Exploration, Preparation, Implementation, Sustainment (EPIS) and Reach, Effectiveness, Adoption, Implementation, Maintenance (RE-AIM) frameworks, we used quantitative and qualitative data to assess program reach, adherence, effectiveness, maintenance, and implementation barriers/facilitators.

**Results:**

Since CPA approval in January 2024, 65 patients were referred. Figure 2 describes quantitative results. Key differences between programs included health system structure, staffing, and LAI ART eligibility criteria. Qualitative interviews with leadership and staff found the program acceptable and feasible, with improvements in overall care coordination, verification of clinical eligibility, and benefits investigation. Persistent barriers included limited clinic capacity and insurance approval.

**Conclusion:**

A pharmacist-led LAI ART model is feasible and sustainable in FQHCs and adaptable to diverse health system and staffing structures. Broader replication, implementation, and evaluation across a larger sample of clinics are warranted.

**Disclosures:**

Jure Baloh, PhD, ViiV Healthcare: Grant/Research Support Gabriella A. Douglass, PharmD, AAHIVP, BCACP, Gilead - FOCUS Partnership: Grant/Research Support Ashlyn Curry, PharmD, AAHIVP, Focus Program - Gilead Sciences: Grant/Research Support Elma Abdullah, PharmD, Gilead/FOCUS: Grant/Research Support

